# Disseminated Peritoneal Leiomyomatosis With Unusual Lung Mesenchymal Malignant Transformation

**DOI:** 10.7759/cureus.31307

**Published:** 2022-11-09

**Authors:** Huda E Buhusayyen, Fatema A Alkhan, Nouf Behzad, Yaser Alderazi, Ghassan Salman

**Affiliations:** 1 Obstetrics and Gynecology, Salmaniya Medical Complex, Manama, BHR; 2 General Surgery, Salmaniya Medical Complex, Manama, BHR; 3 Vascular Surgery, Salmaniya Medical Complex, Manama, BHR

**Keywords:** lung metastasis, mesenchymal transformation, sarcoma, dpl, tamoxifen, postmenopausal, disseminated peritoneal leiomyomatosis

## Abstract

Disseminated peritoneal leiomyomatosis (DPL) is a benign metastasis of leiomyoma mimicking metastasis of malignancy. It usually affects premenopausal women. Malignant transformation is a rare clinical scenario of DPL. However, its etiology is unknown with unusual growth patterns, either of which makes the diagnosis difficult. It was postulated that the pathophysiology of DPL is metaplasia of mesothelial cells under the effect of hormonal stimulation. Hence, we reported the case of a 62-year-old woman with a history of left breast cancer, who presented with DPL and metastasis to the lung with malignant transformation after two years of starting prophylactic tamoxifen therapy. The influence of tamoxifen use on the development of DPL is not fully understood; this is a rare case that highlights a possible association between tamoxifen and the malignancy transformation of DPL. Hence, it may help raise awareness among clinicians dealing with women using tamoxifen or other hormonal therapy, and the risk of DPL development with potential malignant transformation in such patients.

## Introduction

Disseminated peritoneal leiomyomatosis (DPL) is a rare benign disease with an estimated prevalence of <1:1000,000. It was first described by Willson and Peale in 1952 [1]. The pathophysiology of DPL is still not fully understood but the involvement of a complex interaction between genetic predispositions, submesothelial multipotent cell metaplasia, and hormonal stimulation is hypothesized [2,3]. Travassoli and Norris suggested that DPL was abetted by the hormonal effect [4]. The association with pregnancy, oral contraceptive pills use, tamoxifen therapy, and estrogen secretary tumors suggests hyperestrogenic state as a possible factor and hence supports this hypothesis [5,6]. Tamoxifen is a nonsteroidal selective estrogen modulator with an agonist-antagonist effect on estrogen receptors. Tamoxifen is used as an adjuvant, chemoprophylaxis, and palliative therapy in women with breast cancer. The association between tamoxifen use and the acceleration of the growth of uterine leiomyoma as well as the development of new leiomyoma was reported [7,8]. In this report, we describe an unusual malignant transformation of DPL in postmenopausal women, in association with tamoxifen therapy.

## Case presentation

We report here the case of a 62-year-old postmenopausal female, a known case of diabetes, hypertension, G6PD reduced activity, and breast cancer with left-sided mastectomy four years ago; the postoperative patient was kept on prophylaxis tamoxifen; however, the patient was non-compliant and took tamoxifen irregularly for two years. The patient was referred to gynecology oncology after presenting to the emergency room with progressive abdominal distension and pain for one year. CT scan showed bilateral complex pelvic abdominal mass, most likely ovarian with thickening of peritoneal reflection and nodular deposits, multiple left lung pulmonary nodules. The patient had a history of total abdominal hysterectomy 17 years ago due to fibroid. It was unknown if the ovaries were removed or not. Her surgical records and histopathology report were lost. Hence, an ultrasound-guided biopsy was done for the patient to determine the nature of the masses; a core biopsy was obtained from the abdominal nodules, which showed linear fragments of highly hyaline stromal tissue, no atypia or malignant cell was noted, and the pathologist recommended repeating the sample if suspicion still exists.

**Figure 1:**
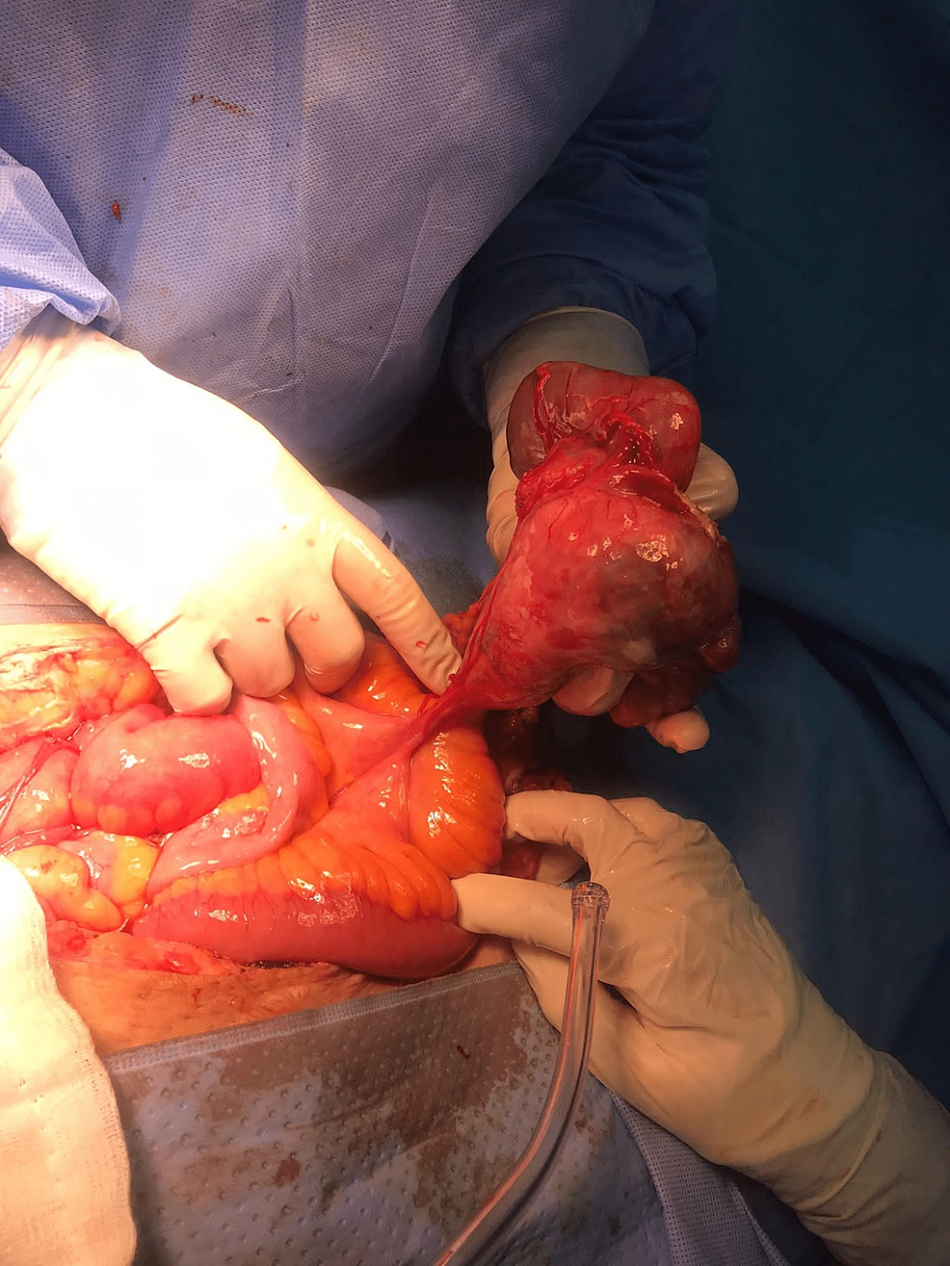
Intraabdominal leiomyoma attached to mesocolon measuring 13 x 12 x 3.5 cm

The patient underwent laparotomy for excision of the pelvic mass. Intraoperatively, a giant free mobile cyst measuring around 28 x 26 cm in size, filled with gelatinous greenish fluid, was found attached to the mesentery; two other masses were also seen, one attached to the omentum measuring 5 x 5 cm, and the other attached to the mesentery measuring around 13 x 12 x 4 cm (Figure 1). A frozen section was sent to the pathological laboratory, grossly the largest cyst was round, tan to yellow, ruptured, measuring 28.5 x 26 x 6.8 cm, the outer surface was nodular, and the cut section showed solid tan nodules, with the largest measuring 10 x 4 x 4 cm. The second cyst was multiloculated with an intact capsule measuring 13 x 12 x 3.5 cm, the outer surface was inked black, the cut section showed a cyst filled with blood, mucus fluid, and multiple solid areas, and the largest area measured 7.5 x 5 x 4 cm. The third section from the omentum showed nodules composed of leiomyomas.

**Figure 2:**
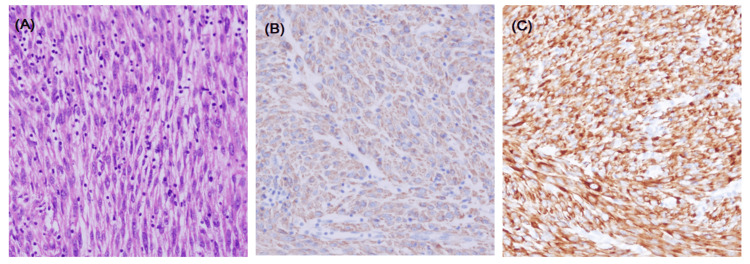
Histological findings of disseminated peritoneal leiomyomatosis tumor. (A) Benign-appearing spindle-shaped cells arranged in intersecting fascicles (hematoxylin-eosin stain; original magnification x200). (B) Immunohistochemically, the tumor was positive for SMA. (C) Immunohistochemically, the tumor was positive for desmin SMA, smooth muscle actin.

Histopathology consisted of relatively encapsulated cellular tumor composed of a fascicle of spindle-shaped cells exhibiting smooth muscle differentiation with mild pleomorphism, and the largest excised mass showed a focal moderate cytological atypia, necrosis, and mitosis (5/10 high-power field [HPF]) with the suspicion of low-grade leiomyosarcoma (LMS). Due to a non-conclusive diagnosis, the specimen was sent to another hospital laboratory for a second opinion. The histopathology report consisted of interlacing bundles of smooth muscle fibers; the cyst that was attached to the mesentery showed a focal increase in mitosis of 4-6/10 HPF and foci of degenerative atypia and no area of convincing necrosis was seen. This is in line with the leiomyoma of the three excised lesions, as the focal area of increased mitosis and degenerative atypia can be seen with leiomyomas. Immunohistochemistry investigation results were positive for SMA, desmin, and estrogen receptors and negative for CD10, CD117, DOG-1, CD34, myogenin, Pan-CK, beta-catenin, and Ki6<10% (Figure 2).

Also, the patient underwent left thoracoscopy and wedge resection of the lung including lower lobe superior segmentectomy + partial lingulaectomy of the left lung. The pathology report consisted of circumscribed neoplasm composed of spindle cells arranged in a fascicular pattern with trapped bronchial tubules lined by respiratory epithelium which was positive for TTF1 and CK7. There were mitosis of 5/10 HPF, focal necrosis, focal nuclear polymorphism, and focal tumor giant cells. All resection margins were free from tumors. Immunohistochemically, the lung specimen was positive for smooth muscle actin (SMA), muscle-specific actin (MSA), desmin, and Ki67:10-12% and negative for CD34, DOG1, and C-Kit beta-catenin. The lung lesion specimen consists of LMS. The case was discussed by the National Tumor Board and the patient opted for chemotherapy in another hospital.

## Discussion

In this report, we present a rare case of DPL with transformation to malignancy in a postmenopausal woman after 17 years of her hysterectomy. Generally, DPL is usually benign; however, the risk of malignant transformation is 2-5% [9]. It usually affects women of reproductive age and it is associated with pregnancy, oral contraceptive pills, tamoxifen use, and estrogen-producing ovarian cancer. The literature shows that it is linked with a state of high estrogen and/or progesterone at the time of diagnosis [6,10,11]. Moreover, hormonal receptors were immunohistochemically stained in a patient with DPL [10-12]. The estrogen receptors were also positive in our case. This can explain the pathogenesis of DPL with a hyperestrogenic state. The woman had received tamoxifen for two years before the symptoms appeared. Tamoxifen has an antiestrogenic and weak estrogenic effect. The estrogenic effect may lead to the rapid growth of leiomyoma. It is interesting in this case that leiomyomatosis has positive estrogen receptors; hence, it may suggest the hormonal influence on the DPL [13]. Unfortunately, the tamoxifen therapy duration to induce DPL and malignancy transformation is not well documented in the literature [14-17]. Controversially, due to her previous history of breast cancer and malignancy transformation of metastatic lung leiomyomatosis, our case may have been a preexisting, yet undiagnosed condition. If this is true, tamoxifen therapy may aid in accelerating a hidden process already in progress.

Rosatiet al. have reported a case series with a patient affected with DPL and malignant transformation and the diameters of the max lesions were in the range of 10-20 cm [18]. As far as we know, our case has the largest diameter of 28.5 cm of DPL reported in the literature.

DPL's resemblance to malignancy by radiology, grossly and intraoperatively, makes its diagnosis challenging [2]. Even microscopically there is some difficulty in the differentiation between DPL and LMS as malignant transformation may occur focally or insidiously, as in our case [19]. However, there are some differences between the cases of DPL and LMS. LMS is characterized by a higher mitotic index, nuclear atypia, and tumor necrosis with infiltration patterns [3]. We found a high mitotic index of 5/10 HPF, nuclear atypia, and necrosis on microscopic evaluation in one of the lung lesions; however, the rest of the pelvic and lung lesions were negative. In addition, in the microscopic evaluation of a patient with DPL, the following are expected to be present: actin, desmin, estrogen, and progesterone receptor. This is in line with our case, where actin, desmin, and estrogen receptors were positive.

DPL with malignant transformation is an extremely rare condition, because of which data on the pathogenesis, risk factors, and management of DPL and its malignant transformation remain insufficient [20].

## Conclusions

In this report, we highlighted a case of DPL with metastasis and malignant transformation in a postmenopausal woman on tamoxifen therapy. Malignant transformation of DPL is a rare, yet critical condition that is best managed by a multidisciplinary team to achieve an optimum diagnosis and management. Tamoxifen indications were expanded worldwide, especially in patients with breast cancer; nevertheless, industrious surveillance of the adverse side effects is needed. Further research studies will be necessary to address if the association between tamoxifen therapy and DPL exists. As such, this case report will create awareness of this rare event and will help in guiding the providers caring for women using tamoxifen or other hormonal therapy. Furthermore, another hormonal therapy as a selective estrogen receptor modulator without the uterine-proliferative effects, such as raloxifene, may be more suitable for women with a previous history of leiomyoma.
